# La biopsia líquida en el manejo del cáncer: una nueva herramienta revolucionaria de la medicina de precisión, aún con limitaciones

**DOI:** 10.1515/almed-2020-0038

**Published:** 2020-05-05

**Authors:** María Arechederra, Matías A. Ávila, Carmen Berasain

**Affiliations:** Programa de Hepatología, CIMA, Universidad de Navarra, Pamplona, España; Instituto de Investigaciones Sanitarias de Navarra-IdiSNA, Pamplona, España; CIBERehd, Instituto de Salud Carlos III, Madrid, España; Programa de Hepatología, CIMA, Universidad de Navarra, Avda. Pio XII, n55. 31008, Pamplona, España

**Keywords:** biomarcadores circulantes, células tumorales circulantes (CTC), ADN tumoral circulante (ctDNA), medicina personalizada, “circuloma” tumoral

## Abstract

El término “biopsia líquida” se emplea en contraposición a la tradicional biopsia “sólida” de tejido. Esta técnica permite analizar y aislar el material tumoral presente en fluidos biológicos, lo cual podría abrir un amplio abanico de usos clínicos en el área de la oncología. Entre los fluidos biológicos se encuentran la sangre, la orina, la saliva, el líquido cefaloraquídeo (CSF), el líquido de derrame pleural o la bilis. En estas muestras biológicas se pueden aislar diversos analitos, de los cuales revisaremos los más relevantes en este trabajo: células tumorales circulantes (CTC), ADN tumoral circulante (ctDNA), proteínas, metabolitos y exosomas. Los biomarcadores que se analizarán dependen del analito, el tipo de tumor y la aplicación clínica, e incluyen mutaciones somáticas, deleciones, amplificaciones, fusiones génicas, marcas de metilación de ADN, miRNA específicos, proteínas y metabolitos. En esta revisión se ofrece una descripción general de las características de los analitos y las diferentes metodologías empleadas para su aislamiento. Así mismo, se describen las aplicaciones de la biopsia líquida en el manejo de los pacientes oncológicos, desde la detección temprana del cáncer a la monitorización de la repuesta a terapia en el cáncer avanzado. Finalmente, también se abordan las limitaciones y cuestiones aún por resolver en relación a esta herramienta.

## Introducción

Incluso antes de que el comité de expertos de la OMS consensuara la definición del término “biomarcador” en Ginebra en 2001 como “cualquier sustancia, estructura o proceso que pueda ser medido en el cuerpo o sus productos e influir y predecir la incidencia de un resultado clínico o una enfermedad” [1], ya se aplicaba la identificación de diferentes biomarcadores en sangre para el diagnóstico y pronóstico de numerosas enfermedades. Algunos ejemplos conocidos son la medición de los niveles de diferentes metabolitos como la glucosa o el colesterol, la presencia de encimas como las transaminasas producidas por la muerte de hepatocitos, o los niveles de inmunoglobulina monoclonal, antígeno carcinoembrionario (CEA, por sus siglas en inglés), alfafetoproteína (AFP), antígeno prostático específico (PSA), antígeno cancerígeno 125 (CA 125) o la gonadotropina coriónica humana (HCG) en el torrente sanguíneo [2, 3]. Todos estos biomarcadores, con diferente sensibilidad y especificidad, se han empleado comúnmente en el diagnóstico de la diabetes, enfermedades hepáticas o la presencia de mieloma, cáncer de colon, hígado, próstata, ovario, o de tumores de células germinales.

Sin embargo, la fiabilidad de estos biomarcadores presentes en fluidos biológicos es limitada [4]. Así mismo, en las últimas décadas, el enorme volumen de datos obtenidos mediante el empleo de técnicas moleculares de alto rendimiento, unido a los avances logrados en el campo de la farmacogenómica, han puesto de manifiesto la importancia de caracterizar el perfil molecular del tumor a la hora de adaptar los regímenes de tratamiento (la llamada “medicina personalizada”), realizar un seguimiento de la respuesta a dicho tratamiento, y detectar la aparición de resistencias [5]. Actualmente, también se sabe que el perfil genético de los tumores es heterogéneo en su localización y dinámico en el tiempo [6, 7].

Las tradicionales biopsias de tejido tumoral presentan ciertas limitaciones como que, en algunos casos, el tumor es de difícil accesibilidad o no se puede realizar dicha biopsia. Además, dada su naturaleza invasiva, las biopsias de tejido tumoral pueden suponer un riesgo para el paciente, lo cual limitará la recogida de muestras longitudinales para el seguimiento l. Por otra parte, las biopsias de tejido pueden presentar un sesgo de muestra, ya que ofrecen un retrato del tumor en un solo punto, lo cual obvia la heterogeneidad genética espacial de los tumores, afectando negativamente a la precisión y sensibilidad de los datos obtenidos [6].

En los últimos años, los esfuerzos de numerosos grupos de investigación y el desarrollo de diferentes tecnologías han permitido mejorar en la detección de material tumoral en fluidos biológicos, impulsando el empleo de metodologías no invasivas para el diagnóstico, pronóstico, seguimiento y selección de terapias para el cáncer [8–13].

**Figura 1:**
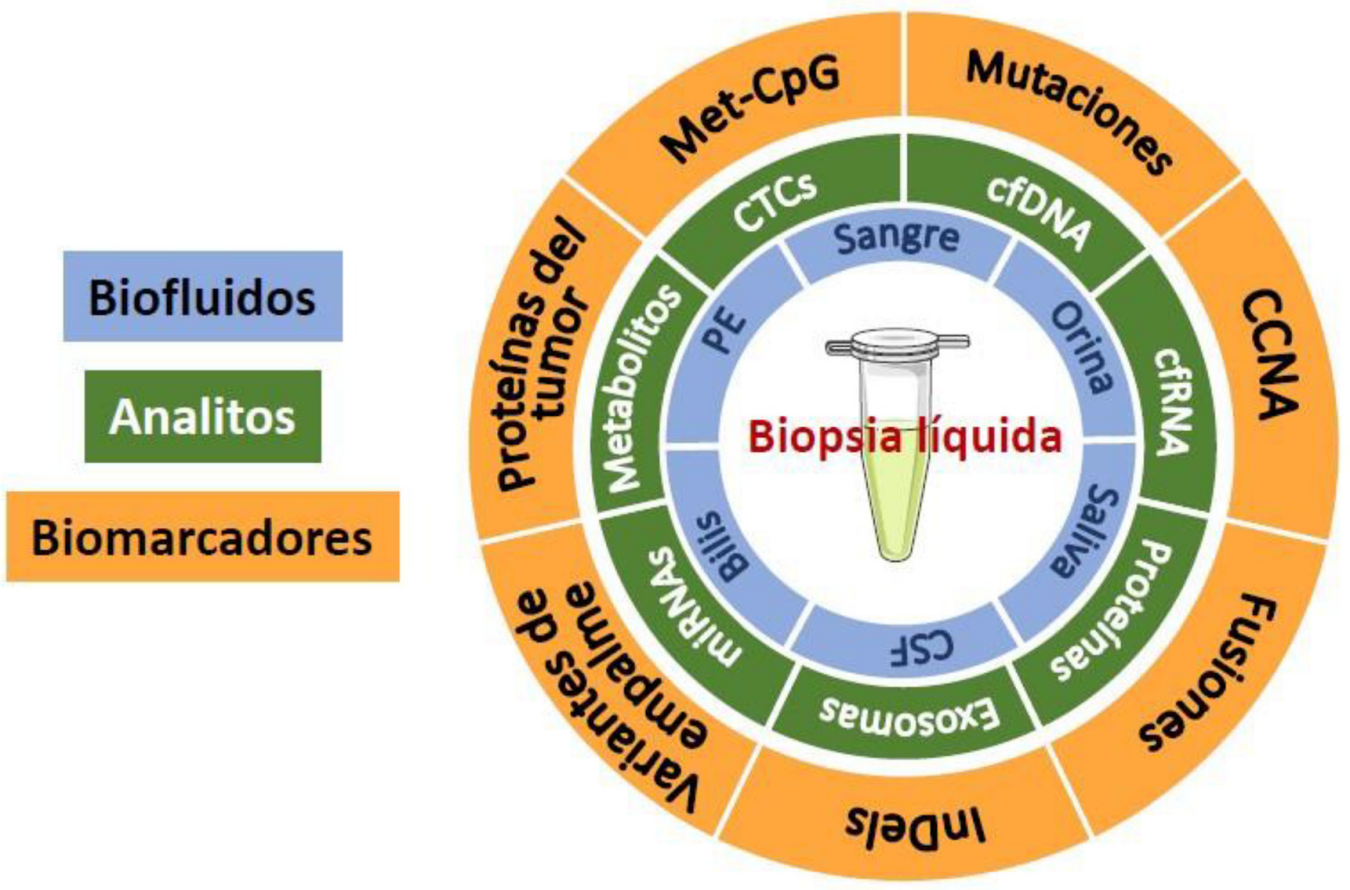
Fluidos biológicos, analitos y biomarcadores que componen la biopsia líquida. Met-CpG: CpGs metilado (dinucleotido C-G). CCNA: número de aberraciones en las copias cromosómicas; InDel: inserciones y deleciones; CTC: células tumorales circulantes; cfDNA: ADN libre circulante; cfRNA: ARN libre de circulante; miRNAs: microRNA; PE: derrame pleural; CSF: líquido cefalorraquídeo.

El término “biopsia líquida” se emplea, por tanto, en contraposición con la tradicional biopsia de tejido tumoral obtenida quirúrgicamente, e implica el análisis de biomarcadores tumorales en material tumoral, por lo general aislado del torrente sanguíneo [13] o de otros fluidos biológicos como la orina, la saliva, el líquido cefalorraquídeo (CSF, por sus siglas en inglés), el derrame pleural o la bilis de pacientes oncológicos [8]. Entre los analitos que se emplean en la biopsia líquida, se encuentran las células tumorales circulantes (CTC), el ADN libre circulante (cfDNA, por sus siglas en inglés), las proteínas, los metabolitos, las vesículas extracelulares y el ARN circulante (cfRNA, por sus siglas en inglés) [10, 14]. Los biomarcadores que se analizan dependen del analito, el tipo de tumor y la aplicación clínica, e incluyen mutaciones somáticas, deleciones, amplificaciones, fusiones génicas, marcas de metilación de ADN, miRNA específicos, proteínas y metabolitos, entre otros (Figura 1). Las tecnologías de identificación y caracterización empleadas dependerán del tipo de analito y del biomarcador [12]. Merece especial mención la revolución de las tecnologías de secuenciación de nueva generación (NGS, por sus siglas en inglés), que han incrementado significativamente el uso de la biopsia líquida basada en el análisis de los ácidos nucleicos [11].

En 2013, la *U.S. Food and Drug Administration* (FDA) aprobó el primer test de biopsia líquida, la plataforma CellSearch® CTC (K073338-FDA), diseñada para el seguimiento del cáncer de mama, colon y próstata metastásicos mediante la medición de CTC [15]. Tres años después, en 2016, se aprobó la primera prueba de biopsia líquida basada en el análisis de cfDNA. Se trata de una prueba diagnóstica complementaria cuyo objeto es la detección de mutaciones del gen EGFR en el ctDNA de pacientes con carcinoma pulmonar no microcítico (NSCLC, por sus siglas en inglés) para que se puedan beneficiar de una terapia dirigida.

No obstante, antes de la plena implantación de las biopsias líquidas en la práctica oncológica, quedan algunas limitaciones por resolver. Además de la optimización y estandarización de protocolos para el aislamiento de analitos, quedan algunas cuestiones por dilucidar, como por ejemplo conocer en profundidad el comportamiento del tumor y su dinámica de diseminación, que de momento dificultan el uso generalizado de la biopsia líquida como nueva herramienta para el manejo del cáncer. En este escenario, y con el fin de acelerar el desarrollo, validación y uso clínico de la biopsia líquida, se ha creado un consorcio de colaboración constituido por entidades públicas, privadas, académicas y agencias reguladores, tanto en Europa (Cancer-ID; https://www.cancer-id.eu/) como en los Estados Unidos (BloodPAC; https://www.bloodpac.org/).

En esta revisión, analizamos los últimos avances en las tecnologías de biopsia líquida empleadas para el aislamiento de analitos y la identificación de biomarcadores, con especial hincapié en las CTC y el cfDNA. Así mismo, se describen las aplicaciones de la biopsia líquida en el manejo de los pacientes oncológicos, desde la detección temprana del cáncer al seguimiento de la respuesta a tratamiento en el cáncer avanzado. Finalmente, también se abordan las limitaciones y cuestiones aún por resolver en relación a esta herramienta.

## Tipos de analitos en los pacientes oncológicos

En los fluidos biológicos de los pacientes oncológicos se pueden detectar diferentes elementos tumorales. Tal como se ha descrito anteriormente, entre estos elementos tumorales se encuentran las CTC, el cfDNA, las proteínas, metabolitos, vesículas extracelulares y ARN libre circulante [10]. No obstante, todos estos analitos tumorales están rodeados de multitud de analitos no tumorales liberados por células normales, lo que dificulta el aislamiento y la detección de la fracción tumoral de interés. En este escenario, el desarrollo de tecnologías de mayor sensibilidad está aumentando el interés en este área, ya que permite a la obtención de material neoplásico por una técnica mínimamente invasiva. Cada analito presenta sus propias ventajas y desventajas, proporcionando información diferente. En algunos casos, su mera detección ya es informativa de por sí, o puede emplearse como material de partida para la detección de biomarcadores. Existe evidencia de que algunos tumores no liberan material tumoral en los fluidos biológicos (20–58%) [17, 18]. Por tanto, los pacientes con estos tumores no se beneficiarían de los avances en las biopsias líquidas y aumentarían el índice de falsos negativos. No obstante, estas aseveraciones deberán ser revisadas una vez se hayan desarrollado técnicas de mayor sensibilidad, ya que su limitada sensibilidad puede estar detrás de la afirmación de que algunos tumores no liberan material tumoral.

Hasta el presente, las pruebas de detección de proteínas tumorales han sido el método de referencia en el cribado y selección de terapias para los diferentes tipos de cáncer. Aunque su uso está muy extendido, su mayor limitación es el sobrediagnóstico [19]. Las investigaciones actuales se centran en la identificación del perfil proteómico en lugar de proteínas concretas para resolver la falta de especificidad [20], así como en mejorar las técnicas de detección [21]. Las vesículas extracelulares tumorales son nanovesículas compuestas por una bicapa lipídica y secretadas por las células tumorales que contienen proteínas y ácidos nucleicos [22]. Estas se pueden hallar en casi todos los fluidos biológicos, especialmente en la sangre. De acuerdo con algunos estudios, sus niveles están elevados en algunos tipos de cáncer [23]. Además, al analizar su contenido, los investigadores han descubierto que los exosomas que contienen la proteína glipicano-1 (GPC1, por sus siglas en inglés) distinguen a los pacientes con cáncer de páncreas con una especificidad y sensibilidad absolutas frente a sujetos sanos [23]. También se han detectado mutaciones de KRAS y p53 [24] o un perfil proteómico específico de microRNA [25] en exosomas de pacientes con cáncer de páncreas. Las células tumorales también liberan diferentes tipos de ARN (mRNAs, miRNAs, lncRNAs) en el torrente sanguíneo. Sin embargo, el ARN es una molécula relativamente inestable, y su detección suele estar asociada a la presencia de vesículas extracelulares. Ya se han publicado algunos ejemplos de biomarcadores exosómicos de ARN [26, 27]. Sin embargo, entre los analitos de las biopsias líquidas, los estudios sobre CTC y cfDNA han experimentado un crecimiento exponencial en las últimas décadas. A continuación se ofrece una revisión detallada de estos dos analitos.

## Células tumorales circulantes (CTC)

Las CTC son una población de células que se han liberado de la masa tumoral y circulan en el torrente sanguíneo. Tenemos que remontarnos 150 años para encontrar la primera descripción documentada de CTC. En 1869, el médico australiano Thomas R. Ashworth observó la presencia de unas células que se parecían a las células del tumor primario en la sangre de un hombre fallecido a causa de un cáncer metastásico [28]. Sin embargo, no fue hasta finales de los años noventa del siglo pasado cuando se produjo el estallido de las CTC, después de que Racila et col. desarrollaran una técnica de enriquecimiento inmunomagnético para la detección de CTC. Los autores, también demostraron entonces la presencia de CTC en los primeros estadios de la enfermedad y señalaron su uso potencial para la monitorización de la respuesta al tratamiento y de las recurrencias [29, 30].

### Fuentes y características de las CTC

La diseminación de las células tumorales en el sistema circulatorio es el primer paso en el proceso metastásico [31]. Aunque los mecanismos implicados en dicho proceso aún están por dilucidar, se sabe que la liberación de CTC en vasos sanguíneos o linfáticos se puede producir mediante la liberación pasiva del tumor primario o a través de un proceso activo asociado a la transición epitelio-mesenquimal (EMT, por sus siglas en inglés) [32]. Una vez en la circulación, si las células sobreviven y se diseminan, se puede desarrollar un tumor secundario en otro órgano [31]. Sin embargo, la metástasis es un proceso enormemente inefectivo, y la mayoría de estas células circulantes mueren debido a traumatismo, estrés oxidativo o los ataques del sistema inmune [33]. Además, la presencia de CTC en la sangre de un paciente oncológico es muy poco frecuente, con 1 CTC por cada 10^6^ – 10^7^ células sanguíneas, dependiendo del estadio de la enfermedad [34] y con una semivida en circulación de menos de 2,5 horas [35]. Las CTC son una población de células heterogénea a nivel genético, transcriptómico, proteómico y metabolómico [36], lo cual podría indicar que proceden de diferentes clones del tumor primario [37]. Las CTC se pueden distinguir de las células sanguíneas mesenquimales por la expresión de una serie de marcadores epiteliales, tales como la molécula de adhesión epitelial celular (EpCam, por sus siglas en inglés) o proteínas de la familia de las citoqueratinas (CK8, CK18 and CK19), o incluso por su morfología epitelial [34]. Sin embargo, tal como se ha descrito anteriormente, y con gran importancia a tener en cuenta en los protocolos de aislamiento, las CTC pueden haber experimentado una transición EMT, perdiendo los marcadores epiteliales y su morfología característica, y expresar en su lugar marcadores de EMT conocidos, como son los componentes de la vía del factor de crecimiento transformante b (TGF, por sus siglas en inglés)-beta, la vimentina, la N-cadherina o el factor de transcripción FOXC1 [32, 38]. Además, un porcentaje de la población de CTC puede adquiere propiedades similares a las de las células madre, expresando marcadores de células madre como ALDH7A1, CD44, y KLF4 [39].

En estudios recientes se han detectado CTC en agregados celulares. Aunque estos son extremadamente raros comparados con las CTC, poseen un gran potencial metastásico [40]. Estos agrupamientos pueden proceder directamente del tumor mediante diseminación pasiva o migración colectiva [40, 41], o bien pueden haberse formado *de novo* en el torrente sanguíneo mediante la agregación de CTC individuales [42]. Los agrupamientos de CTC suelen estar formados por entre 2 y 50 células con fuertes contactos célula-célula. En estos agregados multicelulares, las células tumorales están acompañadas por otras células no tumorales como plaquetas, células inmunes o fibroblastos activados por tumores, lo que podría favorecer la supervivencia de estos agrupamientos de CTC [32, 43]. Además, la proporción de estos agregados puede ser mayor de lo estimado, aumentando durante el proceso de metástasis [44]. Existe evidencia de que la queratina 14 [41], placoglobina [40] y CD44 [42], así como algunos marcadores mesenquimales están sobreexpresados en estos agrupamientos de CTC [32].

### Aislamiento de CTC

En las últimas décadas, se han desarrollado una serie de técnicas para aislar CTC individuales en sangre. Estas tecnologías se basan en las diferencias biológicas o físicas entre las CTC y las células sanguíneas no tumorales. No obstante, aislar CTC sigue siendo complicado debido a que son muy poco frecuentes y a su heterogeneidad [36]. Así, y a pesar de la variedad de tecnologías desarrolladas y probadas, estas aún presentan una serie de limitaciones por resolver. Además del método de aislamiento, también hay que tener en cuenta variables pre-analíticas, como el tipo de tubo de extracción de sangre, el tiempo transcurrido entre la toma de muestras y su procesamiento, o la temperatura de almacenamiento, ya que estos pueden influir en el posterior análisis [45]. De este modo, presentamos una descripción general de los métodos de aislamiento de CTC más conocidos, aunque para una descripción más detallada, recomendamos consultar las revisiones publicadas sobre cada tecnología de aislamiento de CTC [14, 46–49].

#### Aislamiento de CTC en función de sus propiedades biológicas

Los métodos basados en las propiedades biológicas de las CTC se basan en una serie de biomarcadores específicos expresados en la superficie celular de estas células, que permiten su captura en muestras de sangre. La selección positiva de CTC se realiza mediante el reconocimiento de antígenos específicos en la superficie de la célula tumoral, por lo general EpCAM y citoqueratinas (CK8, CK18 y CK19). Sin embargo, tal como se ha mencionado anteriormente, algunas CTC pierden sus marcadores epiteliales, expresando en su lugar marcadores mesenquimales y/o de células madre [32, 38], dando lugar a una muestra de sangre con diversos fenotipos de CTC. Por lo tanto, el sesgo que supone el marcador de selección representa una importante limitación de los métodos de enriquecimiento positivo, ya que producen una captura selectiva de subpoblaciones de CTC específicas, omitiendo otras subpoblaciones. Cabe mencionar también que se ha publicado que la captura basada en el reconocimiento de EpCAM es también útil a la hora de aislar células epiteliales circulantes en pacientes con enfermedades benignas de colon [50], lo que puede por tanto llevar al sobrediagnóstico. La selección negativa de CTC consiste en emplear antígenos que se expresan en las células de sangre periférica pero no en las CTC para así capturar y eliminar las células no tumorales de la muestra, dejando intactas las CTC [51]. Para esta técnica se suele emplear el antígeno CD45. Aunque este método ofrece una pureza menor en comparación con la técnica de enriquecimiento positivo, tiene la ventaja de que se aislarán juntas las diversas subpoblaciones de CTC [51, 52]. Para aplicar el principio de inmunoafinidad se suelen emplear dos dispositivos que consisten en los anticuerpos seleccionados unidos a perlas inmunomagnéticas o a chips microfluídicos. Otra limitación de esta metodología es que la unión de las CTC a la superficie del dispositivo puede dificultar su recuperación y el posterior análisis de eventos moleculares. Actualmente, el sistema CellSearch® (Menarini Silicon Biosystems), que combina el enriquecimiento positivo y negativo, es la única tecnología de CTC aprobada por la FDA. Este sistema sirve para cuantificar la cantidad de CTC en sangre mediante el enriquecimiento basado en CD45-, EpCAM+, y citoqueratinas 8+, 18+, y/o 19+ [15].

#### Aislamiento de CTC en función de sus propiedades físicas

Los métodos basados en las propiedades físicas de las CTC se basan en las diferencias físicas entre estas células y las demás células sanguíneas (mayoritariamente leucocitos) tales como su tamaño, densidad, deformabilidad o carga eléctrica [47]. En principio, las CTC tienen mayor tamaño que las células sanguíneas normales [53]. Las CTC presentan una densidad similar a la de las células sanguíneas nucleadas, quedando entre el plasma y los glóbulos rojos tras la centrifugación. Algunos estudios muestran que las CTC tienen mayor capacidad de deformación que las células normales [54, 55]. Además, también se han utilizado las características eléctricas de las CTC para distinguirlas de las células sanguíneas no tumorales mediante la dielectroforesis [56]. Por último, existen otros métodos de aislamiento de CTC basados en sus características funcionales, como son su capacidad para invadir una matriz de adhesión celular, secretar diferentes proteínas o sobreexpresar la enzima telomerasa [13].

### Utilidad, limitaciones y aspectos importantes de las CTC

A pesar de la existencia de numerosas plataformas para el análisis de CTC y el creciente número de publicaciones en este campo, su traslado a la práctica clínica presenta algunas limitaciones. Hasta el presente, algunos estudios indican que el hecho de aislar CTC y su número por mL de sangre son en sí mismos un biomarcador con posibles implicaciones clínicas. No obstante, actualmente la FDA solo ha autorizado el uso de una plataforma de medición de CTC (CellSearch®) como predictor pronóstico, ya que aporta información fiable sobre la supervivencia libre de enfermedad en el cáncer de mama [44], colon [53] y próstata [56] metastásicos. Aquellos pacientes con enfermedad metastásica y con una proporción de <5 CTC por cada 7.5 mL de sangre tienen mayor probabilidad de presentar mejor respuesta clínica que los que muestran >5 CTC por cada 7.5 mL de sangre [57]. Actualmente se está evaluando la validez clínica de la cuantificación de CTC para seleccionar la primera línea de tratamiento en el cáncer de mama metastásico positivo para receptores hormonales (MATABREAST trial; NCT01710605). Sin embargo, tal como indica Aceto [58], se han observado fluctuaciones en el número de CTC en pacientes en un estadio de la enfermedad, carga total y perfil metastásico comparables, poniendo en cuestión la utilidad clínica de analizar la presencia y abundancia de CTC. Aún es necesario seguir investigando los factores que influyen en la diseminación, dinámicas y eliminación de las CTC para obtener una imagen más completa y desarrollar herramientas más precisas para su uso e interpretación.

No obstante, las CTs también pueden ser el analito inicial para el análisis posterior de biomarcadores. A este respecto, las CTC pueden aportar información proteómica, epigenética, genómica o transcriptómica en tiempo real. Por ejemplo, se ha desarrollado un test para detectar en CTC aisladas la expresión de la proteína AR-V7, una variante del receptor androgénico, para seleccionar el tratamiento en pacientes con cáncer de próstata resistente a la castración [59]. Se ha descrito que los perfiles de metilación de genes supresores de tumores detectados a partir de CTC correlacionan con el potencial metastásico y con un peor pronóstico [60]. Además, recientemente se ha desarrollado un sistema de transferencia de proteínas con microfluidos para evaluar los niveles de ocho proteínas en CTC individuales de pacientes con cáncer de mama positivo para receptores de estrógenos [61].

## ADN libre circulante

En la sangre periférica tanto de sujetos sanos como de pacientes, circulan libremente pequeños fragmentos de ADN. En el caso de los pacientes oncológicos, una fracción de estas moléculas de ADN libre circulante (cfDNA) corresponden a ADN tumoral circulante (ctDNA). El primer hallazgo documentado de ADN soluble en sangre data de 1948 y fue realizado por Mandel y Metais [62]. Sin embargo, hasta 1977 no se estableció una relación entre los niveles séricos de cfDNA en pacientes oncológicos y su respuesta a terapia [63]. Y tuvimos que esperar hasta 1994 para que se descubriera la presencia de una mutación, del gen *KRAS,* en el cfDNA extraído del plasma de pacientes con cáncer de próstata [64].

### Fuentes de cfDNA y características

Los fragmentos de ADN se liberan al torrente sanguíneo a través de la apóptosis celular y otros procesos de muerte celular como la necrosis, la piroptosis o la autofagia, así como mediante la secreción celular activa [65]. El cfDNA puede proceder tanto de ADN nuclear como mitocondrial [66]. Estos fragmentos de cfDNA circulante poseen unos 166 pares de bases, lo cual equivale a la longitud ocupada por un nucleosoma. Una vez en circulación, la eliminación de cfDNA se produce mediante degradación enzimática (desoxirribonucleasa I, la proteasa activadora del factor de coagulación VII y el factor H), excreción renal, y el metabolismo hepático y pancreático [67]. El balance final entre la liberación y la eliminación determina la semivida del cfDNA y, aunque varía según el individuo y su estado patofisiológico, suele oscilar entre 16 minutos y 2,5 horas [67, 68]. Algunos estudios epigenéticos han demostrado que la huella de nucleosomas circulantes y los patrones de metilación de cfDNA de los individuos sanos están fuertemente correlacionados con los de las células linfoides y mieloides [69, 70], lo que señala al sistema hematopoyético como la principal fuente de cfDNA.

En los pacientes oncológicos, una proporción de estas moléculas de cfDNA también procede del tumor primario y de tumores secundarios [64]. Aunque inicialmente se pensaba que un mayor nivel de cfDNA en la sangre de los pacientes con cáncer podría ser un biomarcador de cáncer en sí mismo, muchas otras enfermedades han demostrado causar incrementos de cfDNA similares. A este respecto, hay que tener en cuenta algunos aspectos: a) las concentraciones de cfDNA varían enormemente entre individuos y sus condiciones fisiopatológicas, estando elevado no solo en los pacientes con cáncer avanzado, sino también en otros contextos como en las enfermedades autoinmunes, traumatismos, ejercicio extenuante o embarazo; b) en los tumores en estadio más temprano, la cantidad de cfDNA es muy baja, similar a la de los sujetos sanos [71]; c) la fracción de fragmentos de ctDNA en el total de cfDNA es muy pequeña, oscilando entre el 0,01% y más del 10% en función de la carga tumoral [72] y del metabolismo del tumor [73]. Los fragmentos de ctDNA suelen ser más pequeños que los de cfDNA liberado por células sanas. En un estudio reciente se ha demostrado que estas diferencias en los perfiles de fragmentación no solo podrían usarse en el cribado del cáncer, sino también para determinar el tejido en el que se originó el tumor [74].

### Aislamiento del cfDNA circulante

Aunque se puede obtener cfDNA de diferentes fluidos biológicos, hasta la fecha la mayoría de los estudios se han realizado en sangre extraída de venas periféricas. Sin embargo, aún se desconoce si habrá una mayor proporción de ctDNA y por tanto permitiría aumentar el rendimiento de aislamiento, dependiendo del punto de extracción de sangre. A pesar de la intensificación de las investigaciones y los avances logrados, aún no se han establecido protocolos estandarizados ni para la preparación pre-analítica de las muestras tras la extracción de sangre ni para la purificación de cfDNA [75]. Cabe destacar que las variables inherentes a estos pasos pueden afectar a la calidad del analito, comprometiendo la interpretación del resultado final y la comparabilidad de los estudios.

Se han publicado varios estudios en los que se han comparado varias metodologías paso a paso, cuyos resultados han sido recopilados recientemente en diferentes revisiones [45, 75, 76] (Tabla 1). Aunque se puede analizar el cfDNA tanto en suero como en plasma, es preferible emplear el plasma, con el fin de evitar la contaminación con ADN genómico procedente de la lisis de los glóbulos blancos [77]. Así, la sangre debe extraerse en tubos EDTA, debiendo ser procesada en las primeras 4–6 horas tras la extracción. En caso de que su rápido procesamiento no sea posible, hay que emplear tubos especiales de estabilización celular para evitar la lisis de los leucocitos [78]. Algunos estudios sugieren que un protocolo de centrifugación en dos pasos, primero a baja velocidad y posteriormente a alta velocidad, es el método más efectivo para la separación de plasma y el posterior aislamiento de cfDNA [75]. Un método óptimo de extracción de cfDNA implicaría la purificación de todos los fragmentos de cfDNA evitando el ADN genómico y minimizando la presencia de inhibidores de PCR. Ya existen algunos kits en el mercado para la extracción específica de cfDNA basados en el uso de perlas magnéticas o de columnas de centrifugado con membrana de sílice. Algunos de estos kits son manuales, mientras que otros son sistemas automáticos que minimizan la manipulación de muestras. Aunque se desconocen las variaciones entre los diferentes kits y protocolos, el rendimiento varía según el método de extracción empleado [76]. La concentración de cfDNA se puede determinar mediante el empleo de diferentes metodologías como la fluorometría o técnicas basadas en la PCR [75, 76].

**Tabla 1:** Variables metodológicas en el procesamiento y análisis de cfDNA.

Pasos del protocolo	Variables/consideraciones
1. Extracción de sangre [75]	**Tipo de muestra**: el plasma es preferible al suero para evitar la contaminación con ADN genómico. **Tubo de extracción**: dependerá del tiempo de procesamiento: –Tubos EDTA cuando el plasma se va a procesar en 4-6 horas tras la extracción. –Tubos de estabilización celular cuando no es posible el procesamiento rápido (estable hasta ∼ 7 días).
2. Centrifugación para el aislamiento de plasma [75]	**Protocolo**: se precisa un protocolo con dos centrifugados. Primero a baja velocidad (∼2500 g), luego a alta velocidad (∼14000 g). Aparentemente, la temperatura (temperatura ambiente o 4 °C) no es esencial. * Tras la primera centrifugación, se puede congelar el plasma a -80 °C para su posterior procesamiento.
3. Extracción de cfDNA [75, 76]	**Método:** existen diferentes kits de aislamiento de cfDNA (de Qiagen, Promega, Applied Biosystems, Zymo Research, Norgen Biotek, EpiGenTek, entre otros). Aspectos a tener en cuenta: –**Analito:** extracción de cfDNA o de ácidos nucleicos totales (cfNA) –**Manipulación:** manual o automática –**Tecnología empleada:** Basado en membrana de sílice, partículas o perlas magnéticas, o con columnas, entre otras. *Si se ha congelado el plasma a –8^0^ °CC, el descongelado debe hacerse lentamente en hielo.
4. Cuantificación de cfDNA [75, 76]	**Método**: la cuantificación de cfDNA se puede realizar mediante: –Fluorometría (Qubit, Quantus, Quant-iT PicoGreen assays) –PCR: PCR cuantitativa, PCR digital
5. Análisis de biomarcadores [12, 81]	**Biomarcadores analizados:** mutaciones, amplificaciones, fusiones, metilaciones, entre otros. **Método**: dependiendo de la naturaleza del biomarcador, puede que sea necesario realizar pasos adicionales después del procesamiento (por ejemplo, tratamiento de ADN con bisulfito para la detección de metilación). –Análisis de candidatos específicos (qPCR, Digital PCR, entre otros). –Análisis de amplio espectro: Secuenciación de nueva generación (NGS) (secuenciación dirigida, secuenciación del exoma completo, secuenciación del genoma completo, entre otras).

### Biomarcadores en cfDNA, limitaciones actuales y aspectos importantes

Dado que el cfDNA circulante liberado por las células tumorales conserva las características de la célula de origen, la identificación de alteraciones genéticas y epigenéticas específicas en este analito de biopsia líquida se postula como una herramienta de gran potencial en el manejo del cáncer. Tal como se ha mencionado anteriormente, la muestra de ctDNA reflejaría más fidedignamente las características genéticas y epigenéticas del tumor, que la tradicional biopsia de tejido tumoral, representando la heterogeneidad del tumor. Sin embargo, el análisis de cfDNA en pacientes oncológicos presenta algunas limitaciones. Una de ellas es la baja proporción de fragmentos con mutaciones en la muestra total de cfDNA, lo cual limita la capacidad de detección, dando lugar a falsos negativos [72]. Además, se ha demostrado recientemente que algunas mutaciones pueden producirse durante la hematopoyesis clonal, en cuyo lugar no provendrían de las células tumorales, resultando en falsos positivos [79]. De hecho, según una publicación reciente, un alto porcentaje de las mutaciones de cfDNA halladas tanto en controles como en pacientes oncológicos se produjeron durante la hematopoyesis clonal, lo cual resalta la importancia de procesar simultáneamente cfDNA y ADN extraído de leucocitos del mismo individuo [80]. Por tanto, se debe tener especial cautela a la hora de seleccionar los biomarcadores e interpretar los resultados obtenidos a partir de cfDNA. Las metodologías empleadas para estudiar el material tumoral se pueden dividir en estrategias dirigidas (biomarcadores específicos) y no dirigidas (o de amplio espectro). A la hora de analizar biomarcadores específicos, las tecnologías basadas en la PCR como los sistemas Droplet Digital PCR (ddPCR) y BEAMing (perlas, emulsión, amplificación y magnetismo) han mostrado tener una alta sensibilidad, siendo rápidas y coste-efectivas [81]. Para el análisis de un número variable de biomarcadores, se han diseñado paneles de NGS, como Tam-Seq (secuenciación profunda Tagged AMplicon), Safe-Seq (sistema de secuenciación segura) o CAPP-Seq (caracterización molecular personalizada del cáncer mediante secuenciación profunda) (revisar en [12, 81]). Para identificar nuevos biomarcadores se utilizan tecnologías NGS. NGS tiene la limitación de una menor sensibilidad y de necesitar una mayor cantidad de cfDNA de partida [81]. Además dela enorme cantidad de estudios publicados en este campo y de los esfuerzos realizados por la comunidad científica en las últimas décadas, hasta la fecha la FDA solo ha aprobado tres tests que utilizan cfDNA como analito. Tal como se ha mencionado anteriormente, la primera biopsia líquida basada en cfDNA aprobada fue una prueba diagnóstica complementaria. El test Cobas de mutaciones en EGFR v2 (Roche Molecular System, Inc) se basa en la detección de 42 mutaciones en los exones 18, 19, 20 y 21 en el gen *EGFR* incluyendo L858R, las deleciones del exón 19, y mutación en T790M por PCR en cfDNA extraído de plasma y se lleva a cabo en 4 horas. Este test está aprobado para la identificación de mutaciones en EGFR en pacientes con cáncer de pulmón no microcítico avanzado o metastásico (NSCLC), siendo en caso positivo adecuado para aplicar terapias dirigidas a EGFR [16]. Otra prueba de cfDNA aprobada por la FDA es la prueba Epi proColon (Epigenomics AG) para el cribado del cáncer colorrectal (CRC). Este test está diseñado para la detección mediante PCR en tiempo real de la metilación en el promotor del gen *SEPT9* en cfDNA extraído de plasma tras conversión con bisulfito. El proceso lleva unas 10 horas. Se ha asociado la metilación del promotor de *SEPT9* con el cáncer colorrectal [82]. Para su aplicación directa, las respectivas compañías (Roche Molecular System and Epigenomics AG) han desarrollado kits que incluyen todo el material e instrucciones necesarias para su ejecución, desde cómo preparar la muestra a cómo interpretar los resultados. Mientras que un resultado positivo es diagnóstico y útil para el manejo de los pacientes, un resultado negativo de estas pruebas de biopsia líquida puede no ser concluyente. Finalmente, el kit ClonoSEQ basado en las técnicas de PCR-multiplex y NGS, que fue aprobado por la FDA en septiembre de 2018, se emplea para monitorizar cambios de la carga tumoral a lo largo del tiempo en respuesta al tratamiento o durante la remisión, tanto en pacientes con leucemia linfoblástica como con mieloma múltiple [83]. Estos tests analizan las mutaciones en distintos receptores de inmunoglobulinas y regiones del genoma que suelen mostrar traslocaciones y así llegar a detectar una sola célula cancerígena entre un millón de células [83].

## Aplicaciones y limitaciones actuales de la biopsia líquida en el cáncer

**Figura 2:**
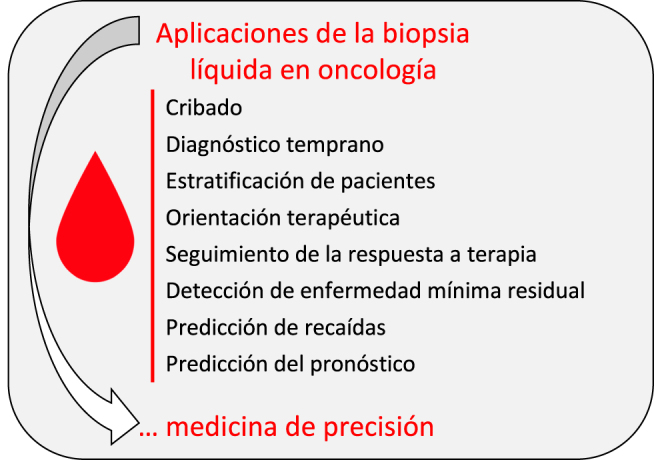
Resumen de las aplicaciones de la biopsia líquida en el manejo del cáncer.

A pesar de los progresos realizados en el manejo del cáncer, esta enfermedad sigue siendo objeto de gran preocupación clínica. Algunas de las dificultades no resueltas siguen siendo el diagnóstico temprano, la estratificación precisa de los pacientes, la selección de tratamientos, el seguimiento de la respuesta al tratamiento y la detección de enfermedad residual mínima y riesgo de recurrencia (Figura 2). Para abordar estos aspectos, las herramientas basadas en las biopsias líquidas están demostrando poseer un gran potencial, habiendo atraído la atención de los inversores, aparte de la de los investigadores [8, 10]. No obstante, aún queda un largo camino por recorrer, ya que muy pocos tests han recibido la autorización o la designación de dispositivo innovador de la FDA (Tablas 2 y 3).

**Tabla 2:** Pruebas de biopsia líquida aprobadas por la FDA.

Kit/test	Compañía	Estado FDA	Alteración detectada/tecnología	Aplicación	Otros datos
TCellSearch Circulating Tumor Cell Kit [15]	Menarini Silicon Biosystems	Aprobado por la FDA (Agosto 2013)	Cuantificación de células tumorales circulantes (CTC) de origen epitelial: Enriquecimiento positivo con EpCAM y detección de citoqueratinas 8, 18, y/o 19.	Pronóstico del cáncer de mama, colorrectal y de próstata	La sensibilidad de este kit dependerá de la positividad de las CTC para el marcador de superficie EpCAM, así como para las citoqueratinas 8, 18, y/o 19.
Epi proColon DNA-methylation blood test [82]	Epigenomics AG	Aprobado por la FDA (Abril 2016)	Detección de citosinas metiladas en el gen *SEPTIN9* en ctDNA mediante PCR a tiempo real.	Cribado del cáncer de colon	La compañía ha desarrollado un kit para evaluar la metilación de *SEPTIN9*, con un tiempo de espera de unas 32 horas. Según la literatura científica, su sensibilidad oscila entre el 69% y el 72%.
Cobas EGFR Mutation Test v2 [16]	Roche Diagnostics	Aprobado por la FDA (Junio 2016)	Detección de 42 mutaciones definidas en el gen del receptor del factor de crecimiento epidérmico (*EGFR*) por PCR a tiempo real	Orientar en la selección de tratamiento en el cáncer de pulmón no microcítico	Este test permite la detección de mutaciones en cfDNA en menos de 4 horas (12). 75% de sensibilidad y 98% de especifidad.
ClonoSEQ [83]	Adaptive Biotechnologies	Aprobado por la FDA (Septiembre 2016)	Detección de reordenamientos de las inmunoglobulinas y regiones que suelen presentar translocaciones mediante PCR-multiplex y secuenciación de alto rendimiento (NGS).	Detección de enfermedad mínima residual en leucemia linfoblástica o en mieloma múltiple	ClonoSeq detecta una sola célula tumoral entre un millón de células. El procesamiento de datos lleva entre 7 y 14 días.

Existen evidencias de que la eficiencia de las terapias antitumorales, incluida la quimioterapia, las terapias dirigidas o los inhibidores de puntos de control inmunitario mejora sustancialmente cuando la carga tumoral es baja, lo cual subraya la importancia de desarrollar métodos de detección temprana. A este respecto, la biopsia líquida es una técnica prometedora para el cribado y diagnóstico temprano del cáncer. Sin embargo, quedan algunas limitaciones por solventar antes de que esta técnica pueda ser incorporada a la práctica clínica [11]. Tal como se ha mencionado anteriormente, uno de los principales obstáculos en el diagnóstico temprano basado en la biopsia líquida es que la presencia de analitos tumorales en los fluidos biológicos de estos pacientes es casi indetectable. Además, aún hay que mejorar en la identificación del órgano de origen y solventar el elevado índice de falsos positivos (sobrediagnóstico) y de falsos negativos (infradiagnóstico) que se producen debido a factores técnicos (selección de biomarcadores, procesamiento de muestras, sensibilidad limitada) y biológicos (tumores no diseminados o hematopoyesis clonal de potencial indeterminado) [11, 17, 79]. A este respecto, la sensibilidad y especificidad de las técnicas podrían aumentar combinando el análisis de distintos biomarcadores en diferentes analitos del mismo paciente [9, 84]. Por ejemplo, con el análisis de sangre CancerSEEK se pueden detectar cuatro tipos comunes de cáncer evaluando en paralelo ocho proteínas y una serie de mutaciones específicas en la misma muestra de sangre [84].

Una vez confirmado el diagnóstico del cáncer, la estratificación del paciente y la selección del tratamiento dependerán de una precisa caracterización molecular del tumor. Así, además de ser mínimamente invasivas, las biopsias líquidas ofrecen la ventaja frente a las biopsias de tejido de ofrecer un retrato más completo del tumor, lo que teóricamente permitirá un análisis más preciso. En este contexto, se están realizando estudios para identificar las mutaciones o patrones de expresión génica susceptibles de ser diana terapéutica. Por ejemplo, la FDA aprobó la prueba Cobas EGFR Mutation test v2 (Roche Molecular System, Inc) para distinguir a los pacientes con NSCLC que se beneficiarán de la terapia dirigida a EGFR [16], mientras que Guardan360 (Guardant Health) examina un panel de 73 genes en cfDNA para facilitar la selección de tratamiento. Del mismo modo, estudios en curso sugieren que los niveles de PD-L1 en exosomas podrían ser predictivos en la terapia anti-PD-1 [85].

Realizar un seguimiento de la respuesta a terapia, así como la detección temprana de las recurrencias es esencial para ganar tiempo y poder administrar rápidamente una segunda terapia oncológica. Las biopsias líquidas también ofrecen la ventaja de permitir la toma de muestras longitudinales para un seguimiento preciso y continuado. Los analitos tumorales deberían disminuir tras la resección quirúrgica total o durante el curso del tratamiento curativo. Si en la fase diagnóstica se identifican biomarcadores tumorales concretos o CTC, estos podrán ser determinados durante el seguimiento y representarán una señal de alarma en el caso de detectarlos. Del mismo modo, algunos estudios demuestran que la detección persistente de analitos tumorales es predictiva de un alto riesgo de recaída en distintos tipos de tumores [72, 86, 87]. El test ClonoSEQ aprobado por la FDA detecta restos residuales de enfermedad en el caso de la leucemia linfoblástica o en el mieloma múltiple tras la finalización de la terapia inicial [83].

**Tabla 3:** Pruebas de biopsia líquida incluidas en el programa *Breakthrough Devices* de la FDA.

Kit/prueba	Compañía	Tecnología/aplicación	Estado FDA
FoundationOne Liquid test	Roche Foundation Medicine	Prueba de NGS para la detección de INDEL, sustituciones, CNV y reordenamientos genéticos seleccionados en 70 oncogenes para el diagnóstico complementario.	Designación de programa *Breakthrough Device* de la FDA (Abril 2018)
Multicancer early detection test	Grail	Prueba de NGS en la que se analizan los patrones de metilación de ctDNA para la detección de diferentes tipos de cáncer	Designación de programa *Breakthrough Device* de la FDA (Mayo 2019)
Guardant 360	Guardant Health	Prueba de ctDNA para la detección de mutaciones (73 genes), amplificaciones (18 genes), fusiones (6 genes), INDEL (23 genes), para orientar en la selección del tratamiento en el carcinoma pulmonar no microcítico.	Designación de programa *Breakthrough Device* de la FDA (Mayo 2019)
Resolution HRD	Resolution Bioscience	Test NGS para la detección de variaciones en las secuencias de los genes asociados a la deficiencia de recombinación homóloga (HRD). Prueba diagnóstica complementaria para el cáncer de próstata.	Designación de programa *Breakthrough Device* de la FDA (Mayo 2019)
CancerSEEK	Thrive Earlier Detection	Test multianalítico que combina la detección de mutaciones en 1.933 locus mediante PCR multiplexado con mediciones de biomarcadores proteicos para el diagnóstico de ocho tipos comunes de cáncer	Designación de programa *Breakthrough Device* de la FDA (Mayo 2019)
ExoDx Prostate IntelliScore (EPI) test	Bio-Techne	Test genómico basado en el análisis de exosomas en orina para el diagnóstico del cáncer de próstata	Designación de programa *Breakthrough Device* de la FDA (Junio 2019)
IvyGeneCORE Test; IvyGene DX Liver Test	Laboratory for Advanced Medicine (LAM)	Hipermetilación de múltiples genes en ctDNA. Para confirmar la presencia de cáncer de mama, colon, hígado y pulmón en estadio temprano	Designación de programa *Breakthrough Device* de la FDA (Septiembre 2019)
[88–90]		*Dispositivo innovador	

## Observaciones finales y cuestiones por resolver

En las últimas décadas, la biopsia líquida se postula como una prometedora herramienta mínimamente invasiva para el manejo del cáncer. El término “biopsia líquida” abarca un amplio abanico de conceptos, ya que incluye conceptos como fluidos biológicos, analitos, biomarcadores, tecnologías y aplicaciones. En estas décadas se están logrando grandes avances y se ha generado nuevo conocimiento que está conformando la base para el desarrollo de pruebas basadas en la biopsia líquida. Sin embargo, la falta de homogeneidad en los protocolos y la competitividad en este campo no permiten afirmar con certeza si estas pruebas de biopsia líquida se podrán incorporar pronto a la práctica clínica. Además, aún quedan algunas cuestiones por resolver. Se han publicado múltiples protocolos, con distintos pasos y condiciones, para aislar los mismos analitos a partir de muestras de biopsia líquida. ¿Es posible desarrollar un único protocolo de aislamiento para cada componente del “circuloma” tumoral? Esto ayudaría a estandarizar e interpretar los resultados. La mayoría de los estudios diagnósticos se han realizado comparando a pacientes oncológicos con controles sanos, aunque en la práctica real, el cribado se realizaría en pacientes con diferentes patologías no oncológicas. ¿Conservarán los test su especificidad calculada? Además, también se han descrito tumores que no liberan analitos a los fluidos biológicos [17, 18]. ¿Existen realmente estos tumores? y, en caso afirmativo, ¿en qué proporción? En caso de que existieran, podría aumentar el número de falsos negativos y complicar así la interpretación de los resultados de estos tests. ¿Cuál es el umbral de sensibilidad de una prueba de biopsia líquida para que ésta sea considerada útil? Con respecto al sobrediagnóstico, antes de considerar válido un test, ¿cuál sería el tamaño muestral y las características de los individuos testados como controles necesarios? A pesar de estas cuestiones y otras que quedan por resolver, los estudios revisados e incluidos en este estudio subrayan el gran potencial de la biopsia líquida en el campo de la medicina de precisión en oncología. Estas cuestiones quedarán pronto resueltas gracias al esfuerzo que están realizando investigadores de todo el mundo por aportar nuevo conocimiento y aplicaciones, lo que permitirá la traslación a la práctica clínica de estas tecnologías mínimamente invasivas que revolucionarán el manejo de los pacientes oncológicos.
